# Efficiency of ODI and APDI of Kim’s cephalometric analysis in a Latin American population with skeletal open bite

**DOI:** 10.1590/2177-6709.24.3.046-054.oar

**Published:** 2019

**Authors:** Paola Janett Caballero-Purizaga, Luis Ernesto Arriola-Guillén, Gustavo Adolfo Watanabe-Kanno

**Affiliations:** 1 Universidad Científica del Sur, Facultad de Odontología, División de Ortodoncia (Lima, Peru).

**Keywords:** Cephalometry, Open bite, Malocclusion, Hispanic Americans

## Abstract

**Objective:**

The objective of this research was to demonstrate the efficiency of the overbite depth indicator (ODI) and the anteroposterior dysplasia indicator (APDI) from Kim’s cephalometric analysis, regarding the determination of the vertical and sagittal patterns of Latin American individuals.

**Methods:**

Two hundred lateral cephalometric radiographs were selected and divided into four study groups, with 50 radiographs each, for carrying out a cross-sectional study. The control group included radiographs of balanced individuals, and the other three groups had lateral cephalometric radiographs of subjects with Class I, II and III malocclusions and with skeletal open bite. After the pilot test was performed to calibrate the investigator, the ODI and APDI were measured. Descriptive statistics were performed and the one-way ANOVA with post-hoc Tukey HSD, or Kruskal-Wallis and Mann-Whitney U-test were used. Also a multiple linear regression was employed.

**Results:**

Statistically significant differences were found for the ODI of all groups (*p*< 0.001), except between Class I group (65.87 ± 4.26) and Class II open bite group (67.19 ± 3.58), both with similar values to each other. For APDI, statistically significant differences were also found for all groups (*p*< 0.001). However, no statistically significant differences were found between the balanced group (83.18 ± 1.71) and Class I group with skeletal open bite (81.78 ± 2.69).

**Conclusions:**

ODI and APDI are reliable indicators to evaluate the sagittal and vertical patterns of an individual, demonstrating their efficiency when a Latin American population was evaluated.

## RESUMO

**Objetivo:**

o objetivo desta pesquisa foi demonstrar a eficácia do indicador de profundidade da sobremordida (ODI) e do indicador de displasia anteroposterior (APDI) da análise cefalométrica de Kim, no que diz respeito à determinação dos padrões vertical e sagital em indivíduos latino-americanos.

**Métodos:**

duzentas telerradiografias em norma lateral foram selecionadas e divididas em quatro grupos, com 50 radiografias cada, para a realização desse estudo transversal. O grupo controle incluiu radiografias de indivíduos com oclusão normal, e os outros três grupos incluíram, respectivamente, radiografias de indivíduos com má oclusão de Classe I, Classe II e Classe III, com mordida aberta esquelética. Após a realização de um estudo piloto para calibração do investigador, fez-se a medição do ODI e do APDI. Foram realizadas estatísticas descritivas e utilizados os testes ANOVA de uma via com *post-hoc* HSD de Tukey, ou teste de Kruskal-Wallis e teste U de Mann-Whitney. Também foi realizada uma regressão linear múltipla.

**Resultados:**

foram encontradas diferenças estatisticamente significativas para o ODI entre todos os grupos (*p*< 0,001), exceto entre os grupos Classe I (65,87 ± 4,26) e Classe II com mordida aberta (67,19 ± 3,58), ambos com valores semelhantes entre si. Para o APDI, também foram encontradas diferenças estatisticamente significativas entre todos os grupos (*p*< 0,001). Entretanto, não foi encontrada nenhuma diferença estatisticamente significativa entre o grupo controle (83,19 ± 1,71) e o grupo Classe I com mordida aberta esquelética (81,78 ± 2,69).

**Conclusões:**

tanto o ODI quanto o APDI são indicadores confiáveis para se avaliar os padrões sagital e vertical de um indivíduo, tendo sua eficácia comprovada para uso na população latino-americana estudada.

## INTRODUCTION

An open bite has been considered as the upper, lower, anterior or posterior lack of teeth contact at the moment of occlusal closure. From an etiological point of view, an open bite can be classified as dental or skeletal. The latter is characterized by an excessive vertical dentoalveolar development on the posterior regions of the dental arches, generating an anteroinferior facial height increase and hyperdivergent maxillaries. This vertical growth can have influence on the treatment complexity and on the high frequency of relapses.^1^

The Multiloop Edgewise Archwire (MEAW) orthodontic philosophy provides efficient and effective results on the skeletal open bite treatment, similar to those obtained with an orthognathic surgery treatment.^2^ ^,^ ^3^ On the other hand, this philosophy is based on an integral diagnostic process using the cephalometric analysis created by Dr. Young H. Kim, who emphasizes the determination of vertical and sagittal growth patterns using the overbite depth indicator (ODI) and the anteroposterior dysplasia indicator (APDI). These indicators offer very important values ​​in relation to the orthodontic treatment planning, mainly when deciding to perform a surgical orthodontic treatment.^4^ ^-^ ^7^

It is very useful for the orthodontist to have at his disposal a simple and efficient method to diagnose a skeletal open bite (ODI) and, at the same time, that may allow him to determine whether a Class I, II or III malocclusion is present in a specific patient (APDI). Unfortunately, most cephalometric analyzes have been performed on Caucasian individuals, whose general characteristics differ from those of other populations. Therefore, standard cephalometric values ​​should be established for each racial group, respecting the craniofacial characteristics of each population. For this reason, several investigations have been carried out in recent years to evaluate and demonstrate the diagnostic efficiency of ODI and APDI indicators on African Americans,^8^ Caucasians,^7^ ^,^ ^9^ ^,^ ^10^ Asians,^4^ ^-^ ^6^ ^,^ ^11^ ^,^ ^12^ and some Latin American groups.^13^ ^-^ ^15^ However, they did not include Class I, II and III pure skeletal open bite groups compared to a balanced group of individuals. This information would help to evaluate the effectiveness of Kim’s cephalometric analysis indicators.

Currently there are no studies accurately reporting on the differential diagnosis of Class I, Class II and Class III malocclusions with skeletal open bite using ODI and APDI, nor studies had determined how affected these patients can be in relation to a balanced patients group. Thus, the purpose of the present study was to evaluate the efficiency of ODI and APDI on the differential diagnosis between Class I, Class II and Class III malocclusions with skeletal open bite and balanced subjects, in a Latin American population, verifying if they are reliable indicators to evaluate the vertical and sagittal patterns.

## MATERIAL AND METHODS 

**Figure 1:**
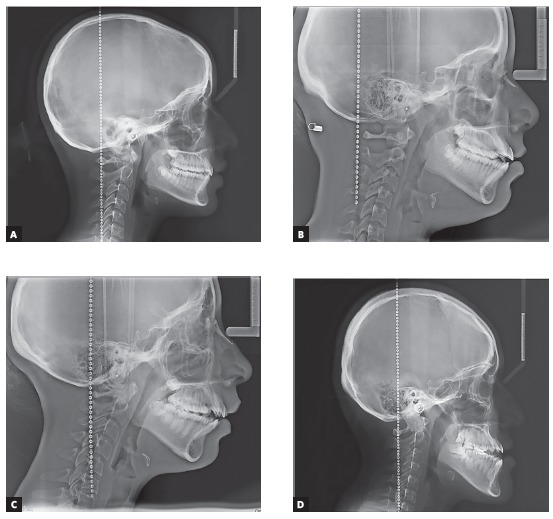
Study groups included on the analysis: A) balanced group; B) skeletal open bite Class I group; C) skeletal open bite Class II group; D) skeletal open bite Class III group.

This cross-sectional study was approved by the research and ethics committee of the School of Stomatology, *Científica del Sur University*, Lima (Peru) under the number 000289. Patients attended IDM Diagnostic Institute during 2013-2016, having an age range between 15 and 40 years. Lateral cephalograms obtained from a total of 1,714 subjects were analyzed. Sample size was calculated considering the comparison of two means using the ODI angular measurement, with a confidence level of 95%, a power of 90%, a variance of 13.03^o^ for the ODI of the control group and a precision of 6.78^o^ (obtained from a preliminary pilot study in which the mean of the ODI of the balanced group *versus* the open bite Class II group was assessed). This estimation showed that a sample of 7 lateral cephalograms was necessary in each group. However, to ensure the validity of comparison among different study groups, sample size was increased to 50 lateral cephalograms (in overall 200 patients) in each of the four groups (Fig 1). 

» Balanced group (n = 50, mean age = 23.66 years, 20 males and 30 females): subjects with skeletal Class I, mesofacial, and with a normal anterior dental relation parameter, including the following cephalometric measurements: ANB = 2 ± 2^o^, USP Projection = between -3 mm and -5.5 mm, FMP = 25 ± 4^o^, overjet = 2.5 ± 2.5 mm and overbite = 2.5 ± 2 mm.
» Class I group with skeletal open bite (n = 50, mean age = 23.32 years, 27 males and 23 females): subjects with ANB = 2 ± 2^o^, USP Projection = between -3 mm and -5.5 mm, FMP =greater than or equal to 30^o^, and overbite = 0 mm or negative.
» Class II group with skeletal open bite (n = 50; mean age = 19.88 years, 16 males and 34 females): subjects with ANB > 4^o^, USP Projection = greater than -3 mm, FMP = greater than or equal to 30^o^, and overbite = 0 mm or negative.
» Class III group with skeletal open bite (n = 50, mean age = 21.82 years, 21 males and 29 females): subjects with ANB < 0°, USP Projection = less than -5.5 mm, FMP = greater than or equal to 30°, and overbite = 0 mm or negative.

Patients under orthodontic or orthopedic treatment, with systemic diseases, with other bone alterations or with prior history of orthognatic surgery were not considered in this investigation.

### Measurements

The images were taken using a digital cephalometric panoramic equipment (ProMax^®^ 2D, Planmeca, Finland), which was set at 16 mA, 84 Kv and 10.9 seconds of exposure. The lateral cephalometric radiographs were taken in maximum intercuspation, with the head on a natural position and with the lips at rest. Radiographs were 1:1 calibrated, and then processed by a calibrated examiner, using the MicroDicom Viewer software. 

**Figure 2:**
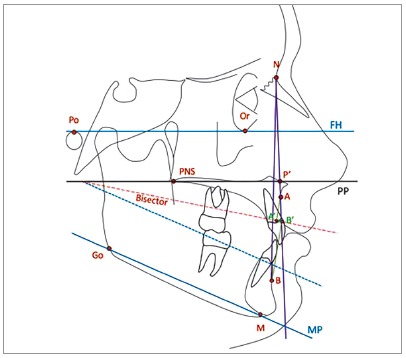
Steiner ANB angle tracing, Tweed FMP angle and USP projection for selecting the sample.

**Figure 3:**
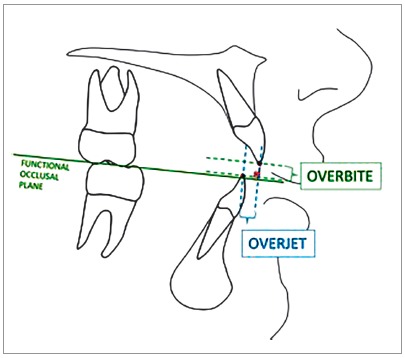
Overbite and overjet tracing for selecting the sample.

The following measurements were performed for the sample selection:

» FMP: From the Tweed cephalometric analysis, angle formed by the Frankfort FH (Po-Or) plane and the mandibular plane (Go-M) (Fig. 2).^16^
» Overbite: Distance between the incisal edges of the upper and lower central incisors, measured perpendicular to the functional occlusal plane (Fig 3).^17^ ^,^ ^18^
» Overjet: Distance between the incisal edges of the upper and lower central incisors measured at the level of the functional occlusal plane (Fig 3).^17^
» ANB: Angle formed by the N-A and N-B planes (Fig 2).^16^
» USP projection: Linear distance between A’ and B’ points, obtained by the orthogonal projection of A and B points to the bisector of the angle formed by the maxillary plane (PNS-P’). P’ is the intersection point of the N-A line with the “p” line (floor of the nostrils, between incisor foramen and ANS) with the mandibular plane (Go-Me).^19^

**Figure 4:**
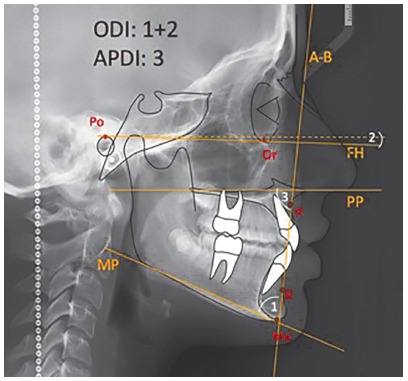
Kim’s analysis of cephalometric points and planes used for ODI and APDI assessment.

The location and layout of the ODI and APDI reference points and planes of Kim’s cephalometric analysis were performed once the lateral cephalograms were selected for the four study groups.^5^ ^-^ ^7^ The ODI was quantified from the arithmetic sum of the angle formed by A-B plane with the mandibular plane (MP), and the angle of the palatine plane (PP) with the Frankfort horizontal plane (FH). A positive value was considered when the palatal plane (PP) was inclined downwards and forward, and the value obtained for the PP-FH angle was added to that of the angle formed by the A-B with the MP plane.^4^ Also, when the palatal plane (PP) was inclined upwards and forward, a negative value was considered and this value was subtracted from that of the angle formed by the A-B plane with the MP plane.^7^ The APDI was quantified from the angle formed between the palatine plane and the A-B plane (Table 1, Fig 4).^13^ ^,^ ^20^ Ten cephalometric tracings were performed per day and all values ​​obtained had an approximation of 0.5 mm or 0.5°.

**Table 1:** Cephalometric points and planes used on Kim’s cephalometric analysis.^7^

Cephalometric points	
Porion (Po)	A point located at the most superior point of the external auditory meatus, which is located on the odontoid process axis and passes through the basion point^17^
Orbitale (Or)	A point located at the lowest point on the infraorbital margin in the middle of the lower boundaries of both orbits^17^
Anterior Nasal Spine (ANS)	A point located at the apex of the anterior nasal spine of the maxilla on the lower margin of the nasal cavity^17^
Posterior Nasal Spine (PNS)	A point located at the posterior limit of the hard palate at the palatine bones junction^19^
Menton (Me)	A point located at the lower and posterior limits of the mental symphysis curvature at the point where the lower border of the symphysis connects the inferior border of the mandibular body^17^
A point (A)	A point located at the greatest depth of the curve formed by the alveolar profile, at the point where it joins the profile of the anterior nasal spine. To locate point A more easily, a line was performed from ANS to the most prominent point of the alveolar ridge in the upper incisor cervical region. Point A was drawn in the deepest part of the alveolar profile in relation to the mentioned line^19^
B point (B)	The deepest point on the anterior curve of the mandibular symphysis. A point located in the deepest part of the alveolo-mental profile on the mental symphysis, in relation to a line tangent to the alveolar border in the cervical region of the lower incisor and to the bony chin prominence^19^
Cephalometric planes	
Frankfort Horizontal Plane (FH)	This plane cross Porion and Orbitale points
Palatal Plane (PP)	This plane cross ANS and PNS points
Mandibular Plane (MP)	Formed by a line that connects the chin to the lower and posterior border of the mandibular body^4^
A-B Plane	This plane cross A and B points

### Calibrations

The examiner was previously trained by a recognized orthodontist specialist on the identification of different cephalometric points and planes used in the present study. Two training sessions were organized prior to the final observations, for calibration of the observer. Intraobserver reliability was assessed by performing ODI and APDI measurements. The observer undertook two viewing sessions, separated by a minimum time interval of four weeks. A pilot study was performed on 20 lateral cephalograms selected from the balanced group, and on 5 lateral cephalograms selected from each of the three skeletal open bite groups. In total, 35 lateral cephalograms were observed to calibrate the observer and to determine the efficiency of the methodology and the sample size. These calibrations were performed using ICC, mean error, Student’s t-test and Dahlberg’s error test - results are shown in Table 2.

**Table 2:** Intra-observer intraclass correlation coefficient.

Measurement	ICC			Mean error	Dahlberg error test	Student t test		p
	Lower limit	Upper limit	ICC			Lower limit	Upper limit	
ODI	0.993	0.998	0.996	0.39	1.02°	-0.2	0.13	0.662
APDI	0.986	0.996	0.993	0.38	1.10°	-0.16	0.15	0.942

ICC= intraclass correlation coefficient.

CI= confidence interval.

### Statistical analysis

The collected data were statistically analyzed using SPSS version 24 for Windows (IBM SPSS, Chicago, IL, USA). Descriptive statistics were used to summarize the ODI and the APDI of Kim’s cephalometric analysis measurements (Table 3). The normality assumption was partially satisfied according to the Shapiro-Wilk test. One-way ANOVA with post-hoc Tukey HSD for independent samples test were used for ODI. When there was no normality (APDI), the Kruskal-Wallis test was performed. In addition, the results of the latter were compared using the Mann Whitney U-test. The *p*-values smaller than 0.05 were considered statistically significant. Finally, two multiple linear regression analyzes were performed to determine the influence of other predictor variables on ODI and APDI.

**Table 3:** Sample descriptive statistics by age and sex.

Groups	n	Age*	Sex**	
		X±SD	Male	Female
Balanced	50	23.66 ± 7.77^a^	20	30
Class I	50	23.32 ± 8.25^ab^	27	23
Class II	50	19.88 ± 5.59^b^	16	34
Class III	50	21.78 ± 6.68a^b^	21	29

* p = 0.035, ANOVA test (different letters are significant, Tukey test).

** p = 0.111, chi square test.

## RESULTS

The sample distribution did not present significant association between sex and the evaluated group (Chi square *p* = 0.111). Likewise, the age did not show significant differences between the groups, except for the comparison between balanced group and open bite Class II group (*p* = 0.044) (Table 3).

The mean value and standard deviation obtained for the ODI of the balanced group (72.10 ± 4.84) ​​were higher than those obtained for the Class I (65.87 ± 4.26), Class II (67.19 ± 3.58) and Class III (60.29 ± 5.23) skeletal open bite groups. Meanwhile, similar values ​​were obtained for the APDI of the balanced group (83.18 ± 1.71) and the Class I group with skeletal open bite (81.78 ± 2.69). These values were lower than those obtained for the Class III skeletal open bite group (87.40 ± 3.08) and higher than those obtained for the Class II with skeletal open bite (73.90 ± 3.46) (Table 4).

**Table 4:** ODI and APDI evaluation in balanced and skeletal open bite Class I, Class II and Class III groups.

		ODI	APDI
Groups	n	X±SD	X±SD
Balanced	50	72.10 ± 4.84	83.18 ± 1.71
Class I	50	65.87 ± 4.26	81.78 ± 2.69
Class II	50	67.19 ± 3.58	73.90 ± 3.46
Class III	50	60.29 ± 5.23	87.40 ± 3.08

Statistically significant differences were found for ODI between all groups, except between Class I and Class II open bite groups, which had similar values. When evaluating APDI, statistically significant differences were found between all groups, except for the balanced group and Class I with skeletal open bite, showing similar values ​​between both groups, as can be seen in Table 5. When multiple linear regression tests were evaluated, FMP and overbite were found to be significant (*p*< 0.05) determining the influence on ODI; for APDI, the ANB angle, USP projection, and sex were found to be significant (*p*< 0.05).

**Table 5:** ODI and APDI values comparison between balanced and skeletal open bite subjects with class I, class II and class III malocclusion.

Compared study groups	Mean difference			95% Confidence Interval	
				Lower Limit	Upper Limit
ODI					
		P_1_	P_2_		
BAL - CI OB	6.236	<0.001	<0.001	3.895	8.578
BAL - CII OB	4.909		<0.001	2.567	7.251
BAL - CIII OB	11.817		<0.001	9.475	14.159
CI OB - CII OB	-1.327		0.458	-3.669	1.015
CI OB - CIII OB	5.58		<0.001	3.239	7.922
CII OB - CIII OB	6.908		<0.001	4.566	9.25
APDI					
		P_3_	P_4_		
BAL - CI OB	1.399	<0.001	0.11	0.503	2.294
BAL - CII OB	9.281		<0.001	8.198	10.364
BAL - CIII OB	-4.215		<0.001	-5.203	-3.227
CI OB - CII OB	7.882		<0.001	6.652	9.113
CI OB - CIII OB	-5.613		<0.001	-6.761	-4.466
CII OB - CIII OB	13.496		<0.001	-14.795	-12.196

P_1_:One-way ANOVA test. P_2_: Tukey HSD test. P_3_:Kruskal-Wallis test. P_4_:Mann-Whitney-U test. OB: open bite; BAL: balanced; CI: Class I; CII: Class II; CIII: Class III.

**Table 6:** Linear Regression analysis for ODI and APDI related to predictor variables.

Measurements	Variables	R^2^	p	Beta	95% Confidence Interval	
					Lower limit	Upper limit
ODI		0.306	<0.001*		70.027	82.487
	Age		0.330	-0.059	-0.151	0.051
	Sex		0.718	-0.022	-1.723	1.189
	FMP		0.012	-0.198	-0.432	-0.053
	Overbite		<0.001	0.407	0.702	1.577
APDI		0.772	<0.001*		79.256	83.405
	Age		0.817	-0.008	-0.060	0.047
	Sex		0.004	-0.100	-1.897	-0.360
	ANB		<0.001	-0.293	-0.891	-0.400
	USP projection		<0.001	-0.570	-1.029	-0.651
	Overjet		0.188	-0.063	-0.361	0.071

## DISCUSSION 

Kim’s cephalometric analysis allows an integral diagnosis of the vertical and sagittal growth patterns of the patient, using ODI and APDI. The main objective of this study was to evaluate the efficiency of Kim’s cephalometric analysis on the differential diagnosis of Class I, Class II and Class III malocclusions with skeletal open bite, and to verify how affected these individuals were in relation to a balanced group. At present, several studies have been carried out on Caucasian,^7^ ^,^ ^9^ ^,^ ^10^ Asian,^4^ ^-^ ^6^ ^,^ ^11^ ^,^ ^12^ Pakistani,^21^ Iraqis,^22^ African American,^8^ and even Latin American populations.^13^ ^-^ ^15^ In these studies, the diagnostic efficiency of this method has been demonstrated. However, no study on the accuracy of differential diagnosis nor comparing subjects with different skeletal open bite malocclusions performed comparisons with a balanced group of subjects.

In order to avoid measurement and sample selection biases, a pilot study was carried out, thus ensuring the reliability of the results. The operator was trained by an orthodontist and the intra-observer variability was performed with a difference of one month. ODI measurement and APDI angles were almost perfect, thus ensuring the measurements reliability. Therefore, one of the strengths of the present study was related to the distribution of the groups in relation to sex and age. Although a difference in age was found between the control group and the open bite Class II group, all cases in this condition were young adults (19.88 ± 5.59 years old) in which the amount of growth is residual - by this reason, the researchers consider that groups were matched.

The results found in the present research demonstrate Kim’s analysis efficiency on the ODI and APDI assessment.^4^ In this study, ODI values ​​for the balanced group were slightly lower than the values ​​found by Kim on Caucasian individuals - the present results were slightly hyperdivergent. This could be due to racial differences in the composition of each sample. Similar results were found by Jones^8^ in an African American sample, by Freudenthaler et al^10^ in Japanese and European, by Kim et al^23^ in Korean individuals, by Saloom^22^ in Iraqis, as well as by Romero^14^ and Castañeda^15^ in Mexicans.

Furthermore, an ODI below the norm can mean a greater possibility of having a skeletal open bite. Conversely, a higher value can indicate a greater tendency for a deep bite. In this regard, the present research clearly shows different ODI values ​​between the balanced group (higher values) and skeletal open bite groups (lower values), independently of the malocclusion, as found by other authors.^4^ ^,^ ^6^ ^,^ ^7^ ^,^ ^9^ ^,^ ^21^ Moreover, the ODI values ​​found in the present study were smaller than 68^o^ for the skeletal open bite groups. This result corroborates Kim proposal,^4^ ^,^ ^6^ which suggests that this measure was diminished on skeletal open bites. This demonstrates that the ODI value is a reliable indicator for the diagnosis of vertical problems, as several investigators verified in other populations.^4^ ^,^ ^6^ ^-^ ^10^ ^,^ ^12^ ^,^ ^14^ ^,^ ^15^ ^,^ ^21^ ^,^ ^24^ On the other hand, the APDI is also considered an excellent parameter for the anteroposterior malocclusions evaluation.^11^ ^,^ ^13^ ^,^ ^25^ ^,^ ^26^ In the present research, APDI values ​​for the balanced group were slightly higher in relation to the values ​​found by Kim and Vietas.^5^ This could be due to racial differences in the composition of each sample, which was corroborated in the present results, similar to those found by Navarrete et al.^13^ and Castañeda^15^ in Latin American groups. However, Oktay^27^ found lower values for the APDI than those found by Kim and Vietas.^5^ A good indicator of sagittal malocclusions should yield different values ​​for skeletal Class I, Class II and Class III malocclusions. In this sense, the APDI value complies with this requirement, since in this study significant differences were found in the groups with different malocclusions. Similar averages to those proposed by Kim were found on Class I group (balanced and with open bite). Class III group were approximately 6 degrees greater in relation to the group with open bite and Class I. Class II group reported values ​​approximately 7 degrees lower than those of Class I with open bite. Similar results were found by different investigators.^9^ ^-^ ^15^ ^,^ ^21^ ^,^ ^23^ ^,^ ^25^ ^,^ ^26^ Meanwhile, as expected, the skeletal open bite Class I group and the balanced group did not present statistically significant differences for APDI values.

In the present study, the multivariate analysis did not show the influence of the sex variable, nor age, except for the APDI, probably due to the fact that in general more women were evaluated in all groups, this distribution could be taken into account for future studies. However, Fatima et al^21^ found no statistically significant differences between the mean values found for ODI and APDI between male and female individuals, nor between subjects in different age groups. Navarrete et al^13^ also reported no statistically significant differences for APDI between genders, and Romero^14^ found that the values of both indicators remained stable during growth.

This reaffirms that ODI and APDI values can be used in a Latin American population. The applicability of these values ​​in different populations demonstrates the universal benefit of its use when evaluating different populations.

## CONCLUSIONS 

ODI and APDI indicators for Kim’s cephalometric analysis demonstrated its efficiency when evaluated in a Latin American population. APDI and ODI are reliable indicators for evaluating an individual’s sagittal and vertical patterns.
